# Functional Outcomes Following Partial Osteotomy of the Calcaneal Tuberosity for Haglund’s Syndrome

**DOI:** 10.7759/cureus.74713

**Published:** 2024-11-29

**Authors:** Saran Karthik S, Pradeep Elangovan, Akshaya Sekar, Praveena Daya A, Vinod Kumar C, Vibishek Raj P, Ashwin VY

**Affiliations:** 1 Orthopedics, All India Institute of Medical Sciences Madurai, Madurai, IND; 2 Orthopedics and Traumatology, Chettinad Hospital and Research Institute, Chennai, IND; 3 Community and Family Medicine, All India Institute of Medical Sciences Madurai, Madurai, IND; 4 Orthopedics, Bhaarath Medical College and Hospitals, Chennai, IND

**Keywords:** american orthopedic foot and ankle society (aofas) ankle /hindfoot score, bursitis, bursitis-surgery, calcaneus-surgery, haglund’s deformity, haglund's syndrome, osteotomy-methods, retrocalcaneal bursitis

## Abstract

Background

Haglund’s syndrome, a common cause of pain in the posterior heel that consists of painful swelling of the local soft tissues and prominence of the posterosuperior calcaneal projection, presents significant challenges in treatment, particularly when conservative management fails. This study evaluates the functional outcomes following oblique partial excision of the posterosuperior portion (calcaneal tuberosity osteotomy) of the calcaneus for Haglund's syndrome.

Methods

A cohort of 30 patients, aged 18 years and older, with persistent heel pain unresponsive to conservative treatments, underwent partial osteotomy using a medial or lateral approach. Patients were assessed pre-operatively and post-operatively at six weeks, three months, and six months using the American Orthopedic Foot and Ankle Society (AOFAS) scoring system. Data were analyzed using descriptive statistics and the Friedman test to evaluate changes in AOFAS scores over time.

Results

The mean AOFAS score improved significantly from 55.17 pre-operatively to 79.27 at six months post-operatively. At six months, 16 (53.3%) participants had AOFAS scores between 80-89, indicating good functional outcomes, while 14 (46.7%) had scores between 70-79. The improvement in functional outcomes was statistically significant (p < 0.001). The study also found both lateral and medial surgical approaches yielded similar results.

Conclusion

Partial osteotomy of the calcaneal tuberosity is a safe and effective surgical intervention for Haglund's syndrome, leading to significant functional improvements. The use of AOFAS scoring provides a reliable assessment of outcomes, confirming the procedure's efficacy.

## Introduction

Haglund's deformity, first described by Swedish orthopedic surgeon Patrick Haglund in 1927 [1], is a bony enlargement on the back of the heel that can irritate the Achilles tendon and bursa, leading to pain. Also known as "pump bump," it is characterized by a prominent bony prominence on the posterosuperior surface of a calcaneus that can cause inflammation of the retrocalcaneal bursa [2,3].

The exact cause of Haglund's deformity is unknown, but several factors can contribute to its development, including repetitive stress on the heel, tight-fitting shoes, and certain foot structures [2,3]. It typically affects middle-aged adults, with women being more susceptible than men. Bilateral involvement is common, and symptoms often include pain at the back of the heel, especially after rest or prolonged activity.

Haglund's deformity can manifest in three ways: posterosuperior calcaneal prominence, retrocalcaneal bursitis, and insertional Achilles tendinopathy [4-9]. Studies suggest that Haglund's deformity and insertional Achilles tendinopathy are often interconnected, with one condition frequently co-existing with the other [7-9]. Pre-existing foot deformities, such as cavovarus, can exacerbate the problems associated with Haglund's deformity [2,3].

Diagnosing Haglund's deformity involves a physical examination and imaging studies. Lateral radiographs of the ankle can reveal the bony prominence in the posterior and superior parts of the tuberosity of the calcaneum. Radiologically, the bursal projection can be confirmed by a Fowler’s angle/posterior calcaneal angle of more than seventy-five degrees, a combination of calcaneal pitch angle and Fowler's angle of more than ninety degrees, and extra bone over the upper parallel pitch line. In some cases, an MRI scan may be necessary to rule out other conditions.

It is important to differentiate Haglund's syndrome from other conditions that can cause similar symptoms, such as traumatic injuries, infectious diseases, and seronegative spondyloarthropathies [10-12].

Fortunately, most cases of Haglund's deformity can be managed conservatively with footwear changes, anti-inflammatory medications, physiotherapy, and, in some cases, casting. If conservative measures fail, surgery may be considered, such as excision of the retrocalcaneal bursa or osteotomy of the calcaneum [9,13-18].

Previous studies have reported varied and inconsistent outcomes for surgical procedures, with some patients experiencing persistent pain and complications [19]. Therefore, this study aims to evaluate the functional outcomes of patients with Haglund's syndrome undergoing surgical treatment in the form of bump resection partial osteotomy of the calcaneal tuberosity.

## Materials and methods

The study was conducted over 18 months at Chettinad Hospital and Research Institute, Chennai, Tamil Nadu, beginning in March 2020 and concluding in September 2021. Ethical approval for this research was granted by the Institutional Human Ethics Committee (IHEC) under approval number 012/IHEC/Feb.2020.

The study employed a cohort design, following a group of patients over time to observe outcomes without manipulating variables. A minimum of 30 participants were recruited, and a convenience sampling technique was adopted. Participants were recruited from the orthopedics outpatient department of the tertiary care teaching hospital who met the inclusion/exclusion criteria and provided informed consent. The inclusion criteria included age 18 years or older, symptoms lasting more than three months, failure to improve symptoms with conservative management, and unilateral or bilateral involvement. The exclusion criteria included age below one year, symptoms lasting less than three months, history of trauma to the affected foot, congenital foot disorders, improvement with conservative management, neurological or musculoskeletal issues in the heel, foot, or ankle, local steroid injections for heel pain within the past six months, diagnosis of seronegative arthropathy, and previous healed fracture of the calcaneus.

Assessment

All the patients underwent pre-operative clinical and radiological evaluation.

Clinical Evaluation

The patient has a history of pain behind the heel, especially during ambulation. Physical examination revealed swelling and tenderness over the posterior calcaneus at the Achilles tendon insertion. Functional assessment was conducted wherein the American Orthopedic Foot & Ankle Society's (AOFAS) hindfoot score was used to assess the function of each foot. A score of 90 to 100 indicates excellent function, 80 to 89 indicates good function, 70 to 79 indicates fair function, and less than 69 indicates poor function.

Radiological Evaluation 

Plain radiograph: Prominence of the posterosuperior calcaneum.

Calcaneal angles: Calcaneal pitch angle (α), Fowler's and Philip's angle (β). The calcaneal pitch angle is between the horizontal plane and a baseline tangent to the medial tuberosity and anterior tubercle. Fowler's and Philip's angle (β) is formed between the baseline tangent line to the medial tuberosity and the anterior tubercle, with the tangent line to the posterior tuberosity and posterior surface of the bursal projection. The normal range is between 44 and 69 degrees. An angle of more than 75 degrees is indicative of Haglund deformity. A total calcaneal angle (α + β) more than 90 degrees is indicative of Haglund deformity.

**Figure 1:**
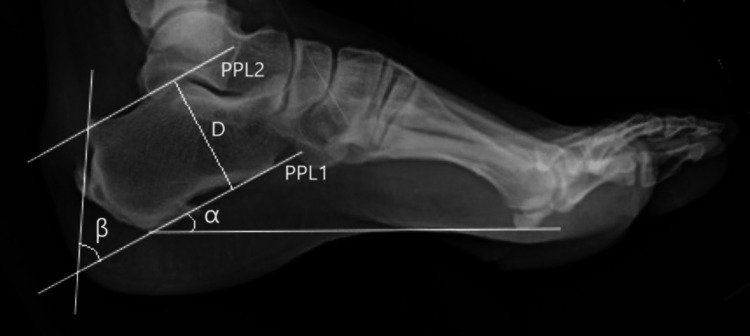
Radiological evaluations Parallel pitch lines (PPL), calcaneal pitch angle (α), and Fowler’s angle (β)

Parallel pitch lines: The lower parallel pitch line (PPL1) is the same as that constructed for Fowler’s angle. A perpendicular line (D) is drawn between the baseline (PPL1) and the talar articulating facet. The upper parallel pitch line (PPL2) is made parallel to the baseline at a distance (D). The part of the bone directly above the upper parallel pitch line (PPL2) is the abnormal bursal projection (Figure 1).

Surgical technique

A partial osteotomy was performed for the study participants. The surgical approach was medial or lateral, and it was determined by symptom laterality and surgeon preference: patients were positioned in a floppy frog lateral position for lateral approaches or supine for medial approaches. All procedures were performed under spinal anesthesia and tourniquet control. The inflamed retrocalcaneal bursa was debrided and excised. A Langenbeck retractor was used to expose the bony deformity, which was removed using an osteotome. Drill holes were placed along the osteotomy site. The wound was irrigated and closed in layers. A below-knee slab was applied with the foot in 20 degrees of plantar flexion. Tendon detachment, reattachment, or splitting techniques were not employed.

Post-operative care

Intravenous antibiotics were administered for two days to prevent infection. A post-operative radiograph was taken to identify whether adequate bone resection was done. For the initial three weeks, weight-bearing is not allowed. After three weeks, the slab was removed, and the patient was started on protected weight bearing with heel raises and exercises to strengthen the calf muscles.

Follow-up

Patients were evaluated clinically at six weeks, three months, and six months (AOFAS scoring was done at every review). A minimum follow-up period of six months was ensured for all participants.

Data was analyzed using IBM SPSS Statistics for Windows, Version 23 (Released 2015; IBM Corp., Armonk, New York, United States). The data was initially entered in Microsoft Excel 2013 (Microsoft Corporation, Redmond, Washington, United States). To understand the distribution of our data, we employed descriptive statistics, which provided frequencies and percentages. Subsequently, a Friedman test was conducted to assess whether there were statistically significant differences between the means of pre-operative AOFAS scores and scores at six weeks, three months, and six months. A p-value less than 0.05 was considered indicative of a statistically significant difference.

## Results

Table 1 represents the distribution of socio-demographic and clinical profiles among the study participants. Fourteen (46.7%) of the participants were in the age group of 41-50 years. Of the participants, 16 (53.3%) were females compared to 14 (46.7%) males. Nineteen (63.3%) of the participants had no comorbidities, while a smaller percentage had conditions like diabetes (10.0%), hypertension (10.0%), or a combination of both (10.0%). The duration of pain was less than one year in 16 (53.3%) participants, and the right side was involved in 18 (60.0%) of the participants. The Fowler's angle was less than 75 degrees in 26 (86.7%) of the participants, and the total calcaneal angle was evenly distributed, with 15 (50.0%) having an angle less than 90 degrees and 15 (50.0%) having an angle greater than 90 degrees.

**Table 1:** Socio-demographic details and clinical profile of the study participants (N=30)

Variables	Frequency	Percentage
Age (in years)		
31-40	11	36.7
41-50	14	46.7
51-60	5	16.6
Gender		
Male	14	46.7
Female	16	53.3
Comorbidities (n=11)		
Nil	19	63.3
Diabetes	3	10.0
Hypertension	3	10.0
Diabetes and hypertension	3	10.0
Anaemia	2	6.7
Duration of pain (in years)		
<1	16	53.3
1-2	10	33.3
>2	4	13.4
Side involved		
Right	18	60
Left	12	40
Fowler’s angle (in degrees)		
<75	26	86.7
>75	4	13.3
Total calcaneal angle (in degrees)		
<90	15	50
>90	15	50

Table 2 depicts the distribution of AOFAS scores among the study participants at different time points: pre-op, six weeks, three months, and six months. At pre-op, all 30 participants (100%) had AOFAS scores below 69, indicating poor functional outcomes before the intervention. At six weeks post-op, 17 (56.7%) of the participants had scores between 70-79, while 13 (43.3%) still had scores below 69. This suggests that some improvement in functional outcomes was observed at this early stage. By three months post-op, the majority of participants, 21 (70%) had scores between 70-79, five (16.4%) had scores between 80-89, and four (13.3%) still had scores below 69. This indicates a continued improvement in functional outcomes compared to the six-week time point. At six months post-op, 16 (53.3%) of the participants had scores between 80-89, and 14 (46.7%) had scores between 70-79. No participants had scores below 69 at this time point. This demonstrates a significant improvement in functional outcomes, with the majority of participants achieving scores in the good to excellent range (AOFAS score 80-100) by six months post-op.

**Table 2:** Distribution of study participants based on pre-op, six weeks, three months, and six months AOFAS score (N=30) AOFAS: American Orthopedic Foot and Ankle Society

Time points	AOFAS Score*				Total
	90-100	80-89	70-79	<69	
	N (%)	N (%)	N (%)	N (%)	N (%)
Pre-op	0	0	0	30 (100)	30 (100)
6 weeks	0	0	17 (56.7)	13 (43.3)	30 (100)
3 months	0	5 (16.7)	21 (70.0)	4 (13.3)	30 (100)
6 months	0	16 (53.3)	14 (46.7)	0	30 (100)

Table 3 represents the changes in AOFAS scores among the study participants at different time points: pre-op, six weeks, three months, and six months. The mean AOFAS score increased significantly from 55.17 (95% CI: 52.820-57.520) at pre-op to 79.27 (95% confidence interval (CI): 77.928-80.612) at six months post-op, indicating a substantial improvement in foot and ankle function over time. The mean rank, which reflects the relative ranking of the scores, also increased progressively from 1.02 at pre-op to 3.97 at six months post-op, further demonstrating the improvement in functional outcomes. The standard deviation (SD) of AOFAS scores dipped from 6.566 pre-operatively to 3.75 at six months post-op. This indicates that the scores became more consistent over time, clustering closer to the mean. The Friedman test showed a highly significant difference in AOFAS scores across the time points (chi-square = 89.141, p < 0.001).

**Table 3:** Comparison of AOFAS score at different time points in all 30 participants (N=30) AOFAS: American Orthopedic Foot and Ankle Society

Time points	Mean	SD	95% Confidence interval		Chi-square	P-value (Friedman test)
			Upper	Lower		
Pre-op	55.17	6.56	52.82	57.52	89.14	<0.01
6 weeks	69.93	4.11	68.45	71.40		
3 months	75.63	5.26	73.74	77.51		
6 months	79.27	3.75	77.92	80.61		

The highly significant p-value (p < 0.001) from the Friedman test confirms that the observed changes in AOFAS scores over time were not due to chance but rather a result of the intervention. This statistical finding further supports the conclusion that the treatment successfully improved the participant's foot and ankle function.

Clinical illustration

Case 1

**Figure 2:**
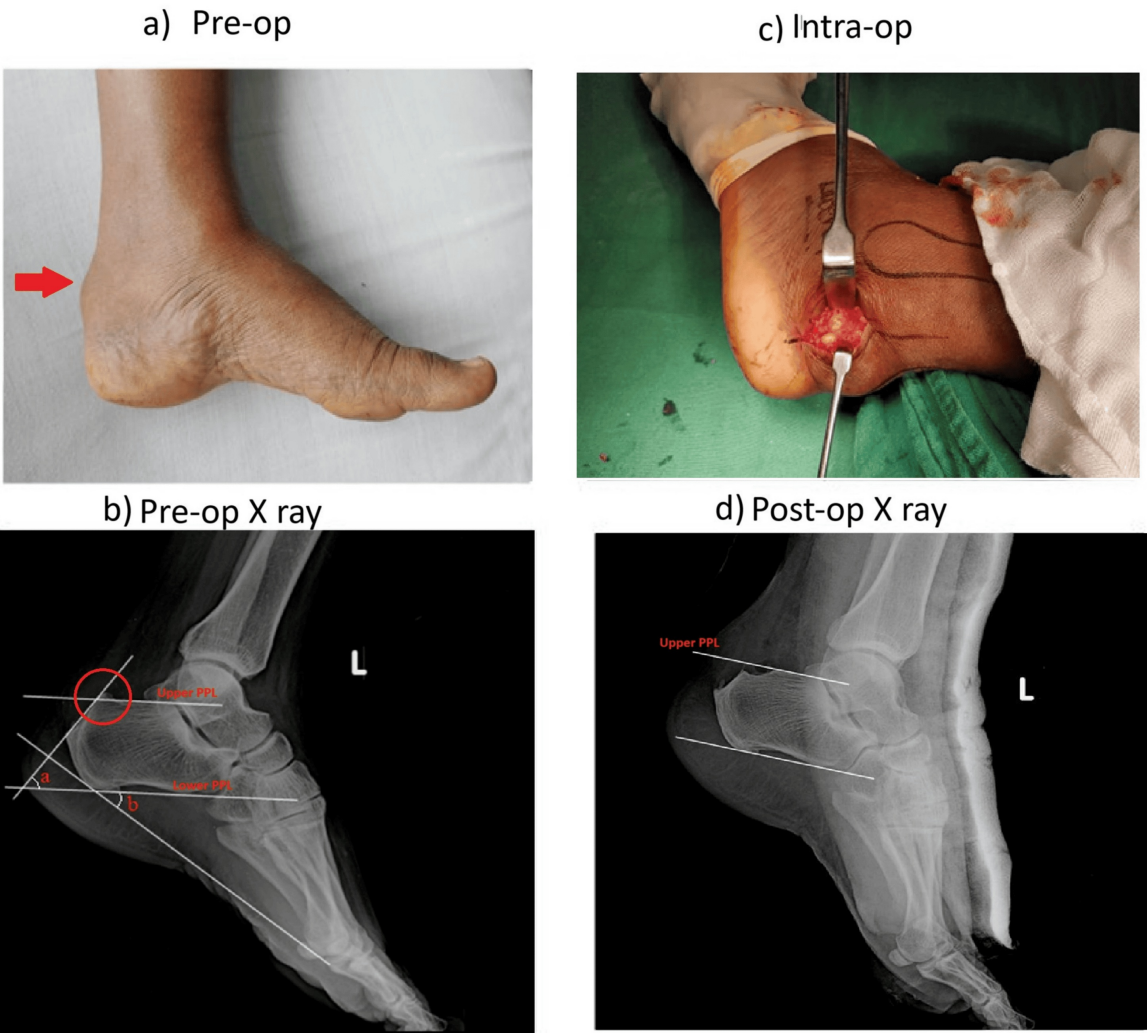
Case 1 details a: pre-op image showing the presence of tender bony prominence at tendo Achilles insertion; b: measurement of Fowler angle (a), calcaneal pitch angle (b), parallel pitch lines (PPL) showing abnormal bursal projection above upper PPL; c: intra-op image showing lateral approach; d: post-operative radiograph showing adequate bone resection below upper parallel pitch line

A 38-year-old woman presented with a two-year history of chronic left heel pain. Despite undergoing physiotherapy and using heel raises and analgesics, her symptoms persisted. Pre-operative radiographic evaluation revealed a Fowler's angle of 54 degrees, a calcaneal pitch angle of 40 degrees, and a total calcaneal angle of 94 degrees. The pre-op AOFAS score was 68. Post-operative AOFAS score at six months improved to 73 (Figure 2).

Case 2

**Figure 3:**
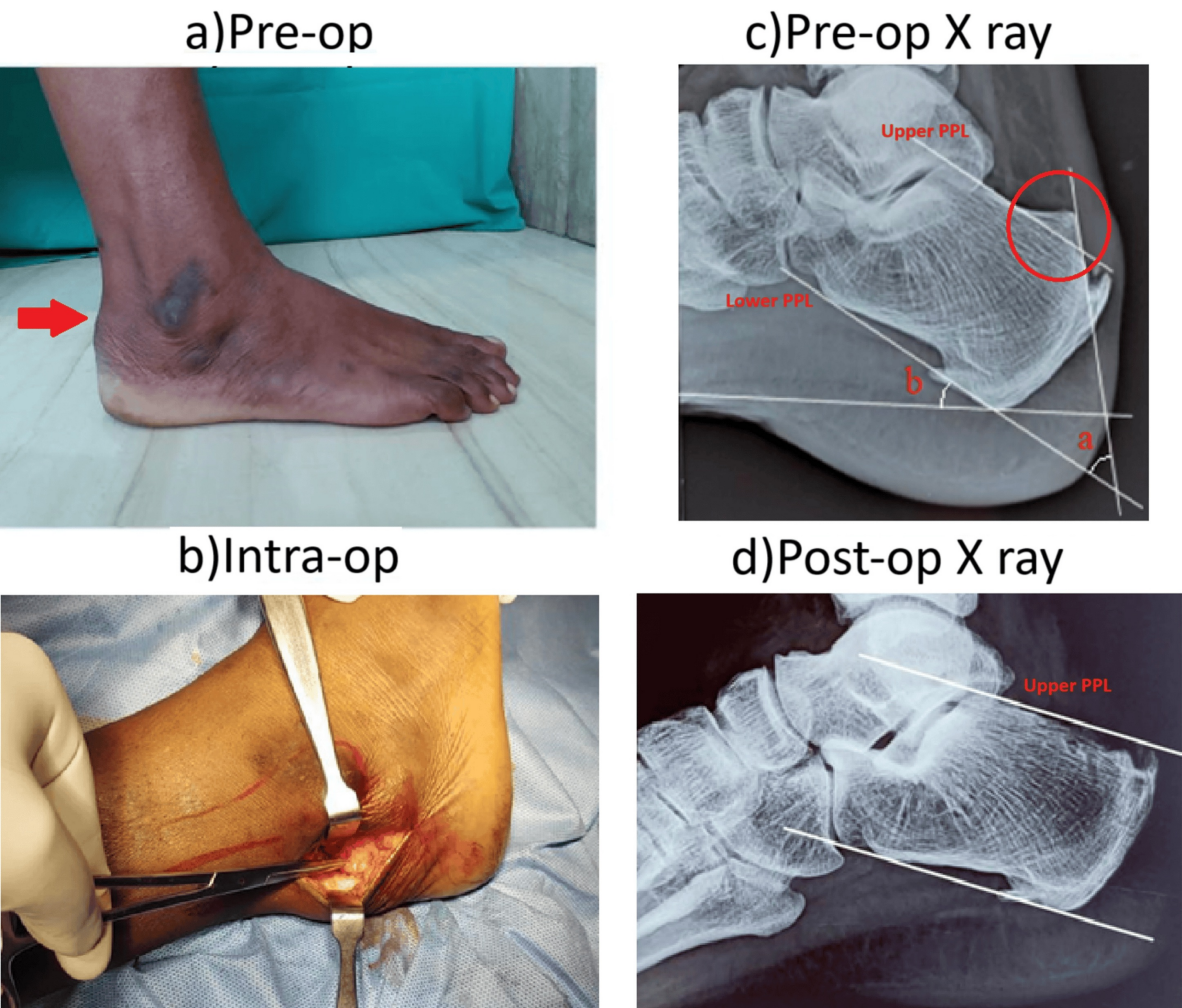
Case 2 details a: pre-op image showing presence of tender bony prominence at tendo Achilles insertion; b: intra-op image showing lateral approach; c: pre-op X-ray showing measurement of Fowler angle (a), calcaneal pitch angle (b), parallel pitch lines (PPL) showing abnormal bursal projection above upper PPL; d: post-op radiograph showing adequate bone resection below upper PPL

A 45-year-old female patient presented with chief complaints of right posterior heel pain for the last year, and six months. Pain is aggravated by walking long distances and on uneven surfaces. No history of recent trauma or injury. The patient was diagnosed with anemia and managed accordingly. Pre-operative radiographic evaluation revealed a Fowler's angle of 50 degrees, a calcaneal pitch angle of 30 degrees, and a total calcaneal angle of 80 degrees (Figure 3). The pre-op AOFAS score was 52. Post-op AOFAS score at six months improved to 83.

## Discussion

On the posterior aspects of the calcaneum, the Achilles tendon gets inserted, which is separated by the retrocalcaneal bursa from the calcaneal tuberosity. Upon ankle dorsiflexion, the pressure on the retrocalcaneal bursa increases, and the tissue is thus prone to inflammation. Haglund syndrome occurs as a result of an “abnormality in the bone and soft tissues of the foot.” An enlargement in the bony section of the posterior part of the heel causes this condition. 

Retrocalcaneal bursitis happens when the bursa lying anterior to the Achilles tendon gets inflamed, adherent, and hypertrophied to the underlying tendon. Such inflammation can be a result of degenerative changes in the tendon. The etiology is not well recognized, but some possible risk factors like hereditary, tight Achilles tendon, and a high arch in the foot have been proposed. It consists of pain in the posterior side of the heel, aggravated during rest. Clinical assessments and X-rays of the ankle are frequently adequate to arrive at a diagnosis of Haglund’s syndrome. The pain may be related to retrocalcaneal bursitis and Achilles tendonitis. Haglund’s syndrome often imitates conditions such as plantar fasciitis, retrocalcaneal bursitis, and seronegative spondyloarthropathies.

Conservative treatment comprises avoidance of tough shoes, activity modification, usage of cushions and pads for heel elevation, and the stretching and strengthening of the soleus and gastrocnemius muscles. Ultrasound therapy, extracorporeal shock wave therapy, and other deep heat modalities could also be tried for symptomatic relief. Steroid injections have also been proposed but carry the risk of rupture of the Achilles tendon. Although conservative measures are often successful, surgical intervention may be required for refractory cases.

Widely accepted surgical techniques for Haglund's deformity are excision of the bursal projection using a medial or lateral approach and dorsal closing wedge osteotomy [19,20]. Of the two, we opted for the excision technique, which was justified by clinical efficacy, cost-effectiveness, and post-operative recovery profiles.

Clinical efficacy

Excision of the bursal projection has demonstrated satisfactory outcomes, with patients reporting significant improvements in pain and function shortly after surgery. Research indicates that this method often results in quicker recovery times and lower complication rates compared to dorsal closing wedge osteotomy, which involves more invasive manipulation of the calcaneus and surrounding tissues, potentially leading to longer rehabilitation periods and higher morbidity.

Cost-effectiveness

Cost considerations also played a crucial role in the choice of surgical technique. The excision procedure was performed at approximately 20,000 INR in a tertiary care setting, significantly less than 50,000 INR [21] associated with dorsal closing wedge osteotomy across various levels of healthcare facilities in India. This affordability enhances accessibility for patients who might otherwise avoid surgical intervention due to financial constraints.

Post-operative recovery

The recovery profile favors excision techniques as they allow for faster mobilization and rehabilitation. In contrast, dorsal closing wedge osteotomy often requires extended periods of immobilization, increasing the risk of complications [22] such as wound dehiscence or infection.

The present study has shown that 14 (46.7%) of the study participants belonged to the 41-50 year age group. Literature has shown that Haglund’s deformity occurs more commonly among middle-aged people [1]. Natarajan S and Narayanan VL's study [19], which involved Haglund deformity-surgical resection by the lateral approach, revealed a similar mean age of 44 years. The present study has shown that 16 (53.3%) of the study participants were females. The ratio of affected females was higher than males. A study done by Stephen MM [9] has shown a similar higher prevalence among females. However, the study stated that most participants were adolescent females, in contrast to the present study, where most of the patients were in the middle age group. 

The present study showed that 18 (60%) of the study participants had right-sided involvement, which is similar to the study done by Anderson JA et al. [18], which also showed bilateral involvement among 6/46 patients, while none of the patients in the present study had bilateral involvement. 

Fowler angles ranging between 44 and 69 degrees are measured as normal, and an angle higher than 75 degrees is connected with prominent posterosuperior calcaneum prominence, indicative of Haglund deformity [23,24]. The present study has shown that Fowler’s angle was more than 75 degrees in four (13.3%) study participants. Similarly, a study by Mir NA et al. [25] showed that three (10.3%) of the 29 study participants had a Fowler angle above 75 degrees. Stephens MM [9] showed that the Fowler angle may not replicate the association of the calcaneus to the sole of the foot. The bursal projection is protuberant in a cavus foot as a result of the vertical calcaneus, even with a normal Fowler angle. 

The present study has shown that the total calcaneal angle was 90.3 ± 5.018. Sharma S et al. [26] highlighted that there was no statistically significant difference in the patient's outcomes according to the total calcaneal angle.

In this study, we used the lateral approach in 16 cases and the medial approach in 14 cases, both of which had similar outcomes with no complications.

The present study found that the crucial part for the effective outcome is the satisfactory resection of the bone, which can permit decompression of the tendon and retrocalcaneal bursa. Inadequate bone excision can result in persistent soft tissue irritation with successive failure of surgical intervention and recurrence.

Further perplexing previous reports of calcaneal osteotomy, the tools used to assess patient outcomes are not standardized, and many have not been validated. In contrast, the present study used standardized and contemporary outcome analysis. The AOFAS score has been previously validated with both internal and external reliability.

The present study has shown that the patients undergoing surgical treatment for Haglund’s deformity have shown fair to good results at the end of six months following oblique bump resection partial osteotomy of the calcaneal tuberosity. The mean AOFAS score during the pre-operative period was 55.17. At six weeks, three months, and six months, the AOFAS score was 69.93, 75.63, and 79.27, respectively, and the results were statistically significant. A similar report has been shown in studies done by Brunner J et al. [19] and Mir NA et al. [25].

Brunner J et al. [19] showed that the mean AOFAS score was 86, with 32 points improvements from the pre-operative period. Anderson JA et al. [17] conducted a retrospective comparative study to assess the effectiveness of the tendon-splitting and lateral approaches for calcaneal tuberosity among patients with Haglund’s syndrome. They showed that the mean AOFAS scores were 43 and 81 in the pre-and post-operative period for the tendon-splitting group and 54 and 86 in the lateral group, respectively.

Natarajan S and Narayanan VL [19] conducted a study to assess the effectiveness of adequate resection of Haglund deformity by lateral approach, which gives a better prognosis. The results showed that the mean AOFAS score during the follow-up was 68, ranging from 55 to 97. Mir NA et al. [25] performed a study to assess osteotomy's functional and clinical outcome through a lateral approach. The results showed that pre-operative and post-operative mean AOFAS scores were 54 and 86, respectively.

Limitations

The primary limitation of this study is its relatively small sample size, short follow-up period (six months), and the absence of a comparison group. To improve the study's validity and generalizability, a multicentric study with a larger sample size, longer follow-up period, and multiple groups comparing different surgical methods would be beneficial. Additionally, incorporating multiple scoring systems to assess functional outcomes could provide more detailed and discrete results, allowing for a more comprehensive understanding of the procedure's effectiveness.

## Conclusions

The functional outcome of patients undergoing oblique bump resection partial osteotomy of calcaneal tuberosity for Haglund’s syndrome is shown to have fair to good results in all patients, which are in concurrence with the previously quoted studies. The procedure showed significant long-term pain relief in most of the patients. Post-operative measurements of parallel pitch lines were identified as a good indicator to assess if the amount of bone to be resected during surgery was either adequate or not.

This study suggests that oblique bump resection partial osteotomy of the calcaneal tuberosity is a safe and effective surgical approach for treating Haglund's syndrome.
